# Epidemiologic and Virologic Investigation of Hand, Foot, and Mouth Disease, Southern Vietnam, 2005

**DOI:** 10.3201/eid1311.070632

**Published:** 2007-11

**Authors:** Phan Van Tu, Nguyen Thi Thanh Thao, David Perera, Khanh Huu Truong, Nguyen Thi Kim Tien, Tang Chi Thuong, Ooi Mong How, Mary Jane Cardosa, Peter Charles McMinn

**Affiliations:** * Pasteur Institute, Ho Chi Minh City, Vietnam; † Universiti Malaysia Sarawak, Kota Samarahan, Sarawak, Malaysia; ‡ Children’s Hospital No. 1, Ho Chi Minh City, Vietnam; § Sibu General Hospital, Sibu, Sarawak, Malaysia; ¶ University of Sydney, Sydney, New South Wales, Australia

**Keywords:** enterovirus 71, viral skin diseases, viral encephalitis, Vietnam, research

## Abstract

During 2005, 764 children were brought to a large children’s hospital in Ho Chi Minh City, Vietnam, with a diagnosis of hand, foot, and mouth disease. All enrolled children had specimens (vesicle fluid, stool, throat swab) collected for enterovirus isolation by cell culture. An enterovirus was isolated from 411 (53.8%) of the specimens: 173 (42.1%) isolates were identified as human enterovirus 71 (HEV71) and 214 (52.1%) as coxsackievirus A16. Of the identified HEV71 infections, 51 (29.5%) were complicated by acute neurologic disease and 3 (1.7%) were fatal. HEV71 was isolated throughout the year, with a period of higher prevalence in October–November. Phylogenetic analysis of 23 HEV71 isolates showed that during the first half of 2005, viruses belonging to 3 subgenogroups, C1, C4, and a previously undescribed subgenogroup, C5, cocirculated in southern Vietnam. In the second half of the year, viruses belonging to subgenogroup C5 predominated during a period of higher HEV71 activity.

## Abstract 2

Human enterovirus 71, but not coxsackievirus A16, is strongly associated with acute neurologic disease.

Hand, foot, and mouth disease (HFMD) is a common febrile illness of early childhood, characterized by 3–4 days of fever and the development of a vesicular enanthem on the buccal mucosa, gums, and palate and a papulovesicular exanthem on the hands, feet, and buttocks (*1*). HFMD is caused by acute enterovirus infections, particularly by viruses belonging to the human enterovirus A (HEVA) species (*1*).

The genus *Enterovirus* of the family *Picornaviridae* is divided into 9 species, 5 of which infect humans. These viruses include the prototype species poliovirus, as well as HEVA, HEVB, HEVC, and HEVD. Viruses belonging to the HEVA species include 11 serotypes of coxsackievirus A (CVA; serotypes 2–8, 10, 12, 14, and 16), and human enterovirus 71 (HEV71) (*2*,*3*).

Although all HEVA viruses can cause HFMD, infection with HEV71 is also associated with a high prevalence of acute neurologic disease (*4*). Despite their close genetic relationship to HEV71, the HEVA CVA viruses rarely cause acute neurologic disease. HEV71 infection is associated with a wide spectrum of acute central nervous system syndromes, including aseptic meningitis, poliomyelitis-like paralysis, brainstem encephalitis, and acute neurogenic pulmonary edema (*4*). Children <5 years of age are particularly susceptible to HEV71-associated acute neurologic disease, which may occasionally cause permanent neurologic disability or death (*4*).

Since the discovery of HEV71 in 1969 (*5*), numerous outbreaks of this infection have occurred throughout the world (*4*). The prevalence of HEV71 infection in the Asia-Pacific region has greatly increased since 1997, concurrent with an increase in the prevalence of HFMD and acute neurologic disease (*6*–*11*). Outbreaks have been recorded in Japan (*12*), Malaysia (*7*), Singapore (*4*), South Korea (*6*), the People’s Republic of China (*13*), and Australia (*14*–*16*). The most extensive epidemic of HEV71 occurred in Taiwan in 1998, with ≈1.3 × 10^5^ cases of HFMD, 405 cases of severe neurologic disease, and 78 deaths. The deaths were due primarily to the development of brainstem encephalitis and neurogenic pulmonary edema (*8*,*17*).

Before 1999, most cases of encephalitis in southern Vietnam occurred in children >5 years of age, of which ≈60% were identified as Japanese encephalitis (diagnostic records of the Pasteur Institute, Ho Chi Minh City, Vietnam). Since 2002, however, viral encephalitis has increasingly been observed in younger children, particularly in those <4 years. Furthermore, since 2002 <27% of encephalitis cases have been confirmed as Japanese encephalitis, which indicates that the epidemiology of viral encephalitis in southern Vietnam may be changing. This situation led us to consider other possible causes for viral encephalitis.

In 2003, we isolated HEV71 (at the Pasteur Institute, Ho Chi Minh City, Vietnam) from 12 patients with encephalitis, who sought treatment at the hospital during an HFMD outbreak in southern Vietnam. To our knowledge, this was the first identification of HEV71 in Vietnam. Although laboratory surveillance has been shown to provide adequate warning of impending outbreaks of HEV71-associated acute neurologic disease (*18*), laboratory surveillance for HEV71 has not yet been established in Vietnam.

## Materials and Methods

### Study Participants and Specimen Collection

Children <15 years of age were admitted to a large pediatric hospital in Ho Chi Minh City, Vietnam. This hospital serves ≈70% of the city’s pediatric population; 764 children with HFMD were enrolled in the study. HFMD was defined as a febrile illness (>37.5°C), accompanied by a papulovesicular rash in a characteristic distribution (oral mucosa, extremities of limbs, buttocks). A total of 1,928 specimens were collected from the children on the day of admission. Each child had at least 1 specimen collected from vesicle fluid, throat swab, or stool. Children who also exhibited acute neurologic disease had a cerebrospinal fluid specimen collected. All specimens were extracted with chloroform (1:10 in phosphate-buffered saline) before virus isolation in cell culture.

### Virus Isolation

Virus isolation was undertaken in cell culture by using both human rhabdomyosarcoma (RD) (ATCC CCL136) and African green monkey kidney (Vero) (ATCC CCL81) cell lines. Each specimen underwent at least 2 cell culture passages in RD and Vero cells before being reported as negative. Samples demonstrating viral cytopathic effect (CPE) were screened for enterovirus RNA by reverse transcription–PCR (RT-PCR), as outlined in the following section.

### RNA Extraction from Cell Culture Supernatants

Total cellular RNA was extracted from cell culture supernatants that demonstrated CPE; Tri-reagent (Ambion, Austin, TX, USA) was used. The RNA obtained from 250 μL of infected cell culture supernatant was suspended in 30 μL RNase-free water and stored at –80°C before use.

### Enterovirus Screening Assays

Cell cultures showing CPE were screened for enterovirus RNA. Two “pan enterovirus” and 1 HEV71-specific RT-PCR assays were used, as described (*19*–*22**).*

#### Pan Enterovirus RT-PCR Assay, 5′ Untranslated Region (UTR)

Briefly, cDNA was prepared in a 10-μL reaction mixture containing 6 μL RNA template, 0.5 mmol/L dNTP, 200 U Moloney murine leukemia virus reverse transcriptase (M-MuLV RT) (Promega, Madison WI, USA), and M-MuLV RT buffer (Promega). cDNA synthesis was performed for 1 h at 42°C. In the PCR step, the 5′UTR was amplified by using 2 μL of cDNA in a 20-μL reaction volume, as described by Romero and Rotbart (*19*). The PCR products were examined by gel electrophoresis. Oligonucleotide primers for this assay (forward primer MD90, reverse primer MD91) flank a conserved nucleotide sequence in the 5′UTR of the enterovirus genome and amplify an expected product size of 154 bp.

#### Pan Enterovirus RT-PCR Assay, VP4

Enterovirus VP4 gene RT-PCR was performed by using primers OL68–1 and MD91, as described (*20*). Briefly, cDNA was prepared from a 10-μL reaction mixture containing 5.5 μL RNA, 0.5 mmol/L dNTP, 200 U M-MuLV RT (Fermentas, Burlington, Ontario, Canada), M-MuLV RT buffer (Fermentas), and the antisense primer OL68-1. cDNA synthesis was performed for 1 h at 37°C. In the PCR step, the VP4 gene was amplified by using 2 μL of cDNA in a 20-μL reaction volume with previously described cycling conditions (*20*).

#### HEV71-specific RT-PCR Assay

The HEV71-specific RT-PCR was performed as described (*21*,*22*) to provide rapid identification of HEV71 in cell culture supernatants that were positive in the screening RT-PCR assay. First, strand cDNA was prepared as outlined above. In the PCR step, the VP1 gene was amplified by using 2 μL of cDNA in a 20-μL reaction volume, as described (*22*). The PCR products were examined by gel electrophoresis. Oligonucleotide primers for this assay (forward primer MAS01S, reverse primer MAS02A) flank a region within the VP1 gene unique to HEV71 and amplify an expected product size of 376 bp.

### RT-PCR for Confirmation and Sequencing

#### HEV71 Complete VP1 RT-PCR Assay

The VP1 gene of 23 HEV71 strains isolated in this study was amplified by RT-PCR by using in-house oligonucleotide primers that flank the entire VP1 gene region, HEV71-VP1-F2 (5′-ATAATAGCAYTRGCGGCAGCCCA-3′; forward) and HEV71-VP1-R1 (5′-TGRGCRGTGGTAGAYGAYAC-3′; reverse). First-strand cDNA synthesis was performed as above, except the reaction was primed with HEV71-VP1-R1. For the PCR step, 2 μL of first-strand cDNA was added to a 50-μL reaction volume containing 1.5 mmol/L MgCl_2_, 1 mmol/L each of primers HEV71-VP1-F2 and HEV71-VP1-R1, 0.3 mM dNTP, 2.5 U Taq DNA polymerase (Fermentas), and Taq polymerase buffer (Fermentas). PCR cycling conditions included an initial denaturation step at 94°C for 5 min, followed by 35 cycles of 94°C for 20 s, 55°C for 30 s, and 72°C for 1 min. This cycling was followed by a final extension at 72°C for 5 min. PCR products (≈1.1 kb) were examined by gel electrophoresis and purified by using the GENECLEAN III kit (Qbiogene, Irvine, CA, USA).

#### Partial VP1 RT-PCR Assay

To identify HEV viruses that were not detected by the VP4 RT-PCR screening assay, a molecular serotyping method based on RT-PCR amplification and sequencing of a portion of the VP1 gene was performed as described (*23*). An ≈340-bp fragment was amplified by RT-PCR by using the forward primer 292 (5′-MIGCIGYIGARACNGG-3′) and reverse primer 222 (5′-CICCIGGIGGIAYRWACAT-3′), under conditions exactly as described by Oberste et al. (*23*). PCR products were examined by gel electrophoresis and purified by using the GENECLEAN III kit (Qbiogene).

### Nucleotide Sequencing of HEV71 VP4 and VP1 Gene Amplicons

Enterovirus VP4 gene amplicons were sequenced on both strands by using the PCR primers. HEV71 VP1 gene amplicons were sequenced on both strands by using the PCR primers and internal VP1 primers 161 and 162, described by Brown et al. (*24*). Sequencing was performed by using the Big Dye Cycle Sequencing kit version 3.0 and an ABI377 automated DNA sequencer (Applied Biosystems, Foster City, CA, USA). The SeqMan software module in the Lasergene suite of programs (DNASTAR, Madison, WI, USA) was used to format the nucleotide sequences. Partial VP1 and VP4 sequences for 173 HEV71 strains and 214 CVA16 strains have been submitted to the European Molecular Biology Laboratory database (partial VP1 gene accession nos. EU072122-EU072195; VP4 gene accession nos. EU051005-EU051317).

### HEV71 VP1 Gene Nucleotide Sequence Data from GenBank

In addition to 23 VP1 gene sequences from HEV71 strains isolated in Vietnam, 26 VP1 gene nucleotide sequences of HEV71 strains available in the GenBank database were included in this analysis, allowing the generation of a dendrogram containing 49 strains isolated between 1970 and 2005 (Table 1). The strains used to reproduce the HEV71 tripartite genogroup structure identified by Brown et al. (*24*) were isolated in the United States, Japan, Australia, Malaysia, Singapore, Taiwan, the People’s Republic of China, Hungary, South Korea, and the United Kingdom.

**Table 1:** HEV71 VP1 gene nucleotide sequences used in reconstruction of the HEV71 dendrograms*

Isolate	Source	GenBank accession no.
CVA16-G10	GenBank	NC_001612
BrCr-CA/USA/70	GenBank	U22521
shzh02–62	GenBank	AY895136
shzh04–12	GenBank	AY895144
shzh01–3	GenBank	AY895132
shzh04–3	GenBank	AY895142
shzh01–4	GenBank	AY895134
SB12007-SAR-03	GenBank	AY905548
SB12282-SAR-03	GenBank	AY905546
SB9465-SAR-03	GenBank	AY258302
SB9508-SAR-03	GenBank	AY258301
S10822/SAR/98	GenBank	AF376079
2037-MD/USA/95	GenBank	AF009556
S18191/SAR/02	GenBank	AY189154
1M/AUS/12/00	GenBank	AF376098
13/KOR/00	GenBank	AY125976
06/KOR/00	GenBank	AY125970
8M/AUS/6//99	GenBank	AF376109
1245a/TWN/98	GenBank	AF176044
3799/SIN/98	GenBank	AF376117
MY104–9/SAR/97	GenBank	AF376072
2027/SIN/01	GenBank	AF376111
SB2864/SAR/00	GenBank	AF376066
8102-WA/USA/87	GenBank	AF009526
7423-MS/USA/87	GenBank	U22522
2229-NY/USA/76	GenBank	AF135868
1011-ND/USA/79	GenBank	AF135864
1001V/VNM/05	This study	AM490141
1089T/VNM/05	This study	AM490142
1091S/VNM/05	This study	AM490143
1129V/VNM/05	This study	AM490144
1135T/VNM/05	This study	AM490145
1177T/VNM/05	This study	AM490146
1192S/VNM/05	This study	AM490147
1277S/VNM/05	This study	AM490148
1301V/VNM/05	This study	AM490149
1303S/VNM/05	This study	AM490150
540V/VNM/05	This study	AM490151
559S/VNM/05	This study	AM490152
666T/VNM/05	This study	AM490153
707V/VNM/05	This study	AM490154
718T/VNM/05	This study	AM490155
730T/VNM/05	This study	AM490156
777T/VNM/05	This study	AM490157
784S/VNM/05	This study	AM490158
900S/VNM/05	This study	AM490159
926V/VNM/05	This study	AM490160
933V/VNM/05	This study	AM490161
962T/VNM/05	This study	AM490162
999T/VNM/05	This study	AM490163

*HEV71, human enterovirus 71; CVA16, coxsackievirus A16.

### Phylogenetic Analysis

VP1 and VP4 gene sequences were subjected to nucleotide-nucleotide BLAST analysis (blastn) by using the online server at the National Center for Biotechnology Information (www.ncbi.nlm.nih.gov/blast). Alignment of the 23 HEV71 complete VP1 gene sequences was undertaken by using the ClustalW program (*25*). A dendrogram was constructed by using the neighbor-joining method with PHYLIP version 3.5 (*26*) and drawn by using TreeView (*27*). Bootstrap analysis with 1,000 pseudoreplicates was performed by using the program Seqboot (*28*). Coxsackievirus A16 (CVA16), strain G10 (*29*), was used as an outgroup in the analysis.

### Statistical Methods

Differences between proportions were tested by using the χ^2^ test with Yates correction or Fisher exact test. Epi Info version 6 (Centers for Disease Control and Prevention, Atlanta, GA, USA) was used for the analysis.

## Results

### Virus isolation from HFMD Patients

An enterovirus was isolated from 411 (53.8%) of the 764 HFMD patients enrolled in the study. The number of CVA16, HEV71, and other enterovirus serotypes isolated from HFMD patients is presented in Table 2. CVA16 was identified in 214 (52.1%) and HEV71 in 173 (42.1%) of the enterovirus-positive HFMD patients. Twenty-four (5.8%) enteroviruses of another serotype were also isolated from HFMD patients (Table 2).

**Table 2:** Total number of enterovirus serotypes isolated from hand, foot, and mouth disease cases, southern Vietnam, 2005

Virus serotype*	No. cases
CVA16	214
HEV71	173
Other enterovirus	24
Negative	352
Total	763

*CVA16, coxsackievirus A16; HEV71, human enterovirus 71.

**Figure 1:**
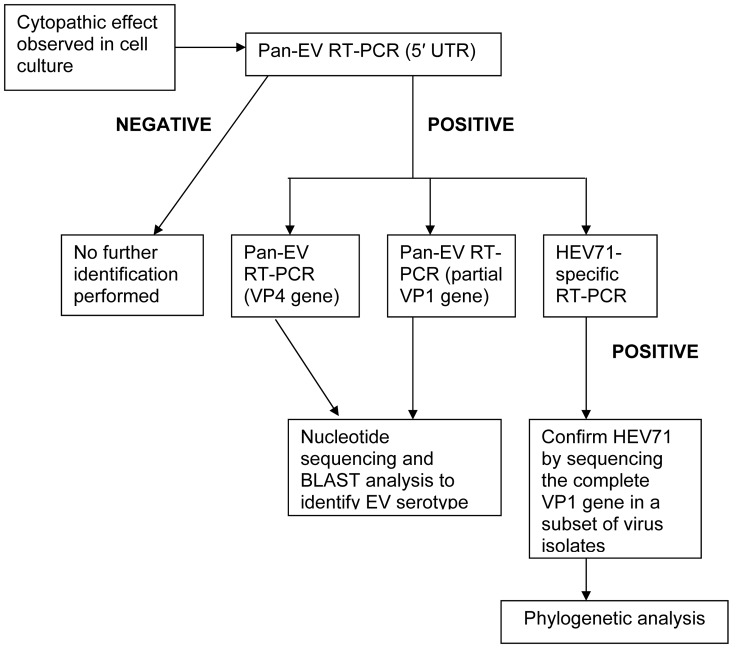
Flowchart showing the procedures used for isolating and identifying enterovirus strains cultured from clinical specimens obtained from children admitted to a large pediatric hospital in Ho Chi Minh City, Vietnam, with a diagnosis of hand, foot, and mouth disease (HFMD) during 2005 and enrolled in this study. EV, enterovirus; RT-PCR, reverse transcription–PCR; 5′ UTR, 5′ untranslated region; HEV71, human enterovirus 71.

Procedures for the isolation and identification of enterovirus strains obtained in the study are presented in a flowchart (Figure 1). Of the 411 enteroviruses isolated in this study, 170 were identified by using HEV71-specific primers. Another 3 were identified as HEV71 when the VP4 and partial VP1 RT-PCR products were sequenced. We used the RT-PCR assay and sequencing of the VP4 gene as a screening tool because a single set of primers allowed us to obtain a preliminary identification of HEV71 or CVA16. In our laboratory, 256 enterovirus isolates were sequenced in both VP1 and VP4, and 100% concordance was found between the VP1 and VP4 results for HEV71 (130 isolates) and CVA16 (61 isolates); only 28 (43%) of 65 other enteroviruses had concordant results in both the VP1 and VP4 sequences (unpub. data). Thus, 24 non-HEV71, non-CVA16 isolates were identified as other enteroviruses.

### Clinical Features of HFMD

**Figure 2:**
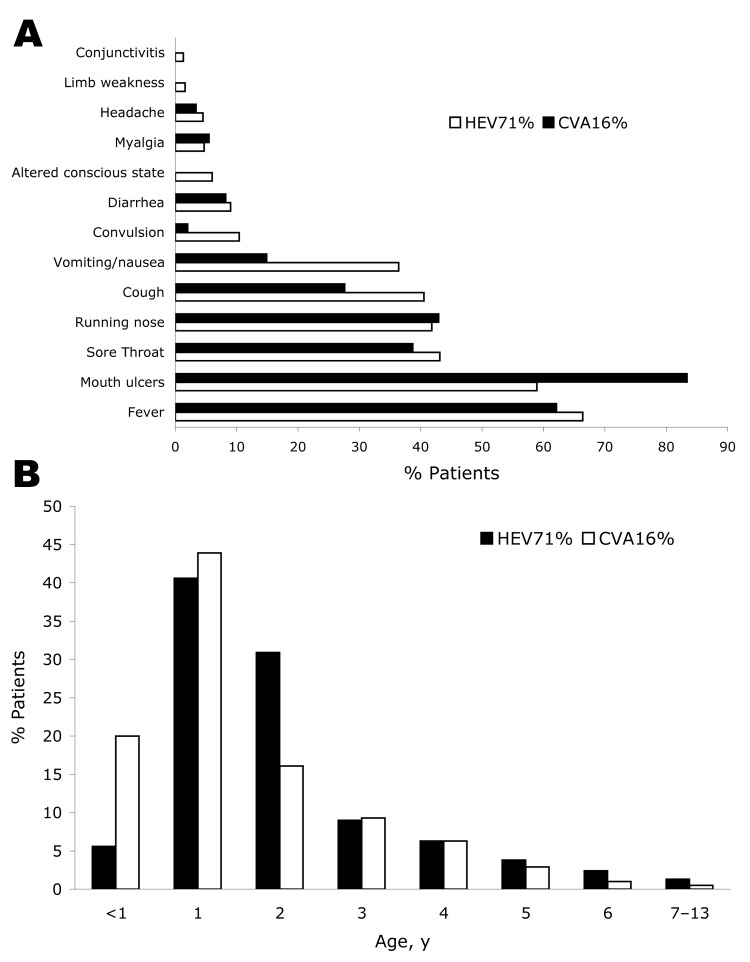
Clinical features of hand, foot, and mouth disease (HFMD) in children admitted to hospital in southern Vietnam during 2005. Features were associated with the isolation of coxsackievirus A16 (CVA16) (214 cases) or human enterovirus 71 (HEV71) (173 cases) from vesicle, throat swab, or stool specimens. A) Percentage distribution of clinical signs and symptoms among identified cases of HFMD. B) Percentage age distribution of patients with identified cases of HFMD.

The clinical features observed in HFMD patients enrolled in the study are presented in Figure 2, panel A. By definition, children enrolled in the study all displayed the characteristic papulovesicular rash of HFMD; 214 cases of HFMD were associated with CVA16 infection, and 173 cases were associated with HEV71 infection. Notably, the formation of ulcers on the oral cavity was observed less frequently with HEV71 infection than CVA16 infection (102 [58.9%] of 173 HEV71 patients vs.178 of 214 CVA16 patients [83.2%]; p<0.0001, odds ratio [OR] 0.29, 95% confidence interval [CI] 0.18–0.48). Cough was also observed more frequently with HEV71 infection than CVA16 infection (70 of 173 [40.5%] vs. 59 [27.6%] of 214; OR 1.79, 95% CI 1.14–2.8). Altered sensorium was experienced by 10 (5.8%) of the 173 HEV71 patients and, as expected, by none of the CVA16 patients. This finding was significant (p = 0.0003), but due to the small numbers, the OR could not be calculated.

Clinical signs of neurologic infection were observed primarily with HEV71-associated HFMD. Convulsions were observed for 18 (10.4%) of 173 HEV71 patients and 4 of 214 (1.9%) CVA16 patients, respectively (p = 0.0007, OR 6.10, 95% CI 1.95–25.15). Vomiting was also significantly more frequent for HEV71 patients (63 [36.4%] of 173) than for CVA16 patients (30 [14.0%] of 214; p value <0.0001, OR 3.51, 95% CI 2.08–5.94). Only patients with HEV71-associated HFMD had alteration of consciousness (10 [5.8%] of 173, p = 0.0003) or limb weakness (3 of 173 [1.7%]). In all, acute neurologic disease accounted for 29.5% (51/173) of identified cases of HEV71-associated HFMD. The case-fatality rate for HEV71-associated acute neurologic disease was 5.9% (3/51) and for all HEV71-associated HFMD was 1.7% (3/173). No fatal cases of CVA16-associated HFMD were identified.

Other clinical signs and symptoms did not differ significantly between HEV71 and CVA16 patients. Sore throat (43.1% of HEV71 patients and 38.7% of CVA16 patients) and runny nose (41.8% of HEV71 patients and 42.9% of CVA16 patients) were observed in approximately half of the HFMD patients. A smaller number of HFMD patients exhibited symptoms of gastrointestinal disorder, such as diarrhea (9.0% of HEV71 patients and 8.2% of CVA16 patients). Myalgia (4.7% of HEV71 patients and 5.5% of CVA16 patients) and headache (4.5% of HEV71 and 3.4% of CVA16 patients) were less common symptoms.

The HFMD cases observed in southern Vietnam occurred primarily in children <5 years of age (Figure 2, panel B). Most HEV71 (136/173 patients) and CVA16 (171/214 patients, 79.9%) infections were identified in children <3 years of age; the peak age-specific incidence of HEV71 (71/173 patients, 40.5%) and CVA16 (94/214 patients [43.9%) infections were identified in children 1–2 years of age.

### Epidemiology of HFMD

**Figure 3:**
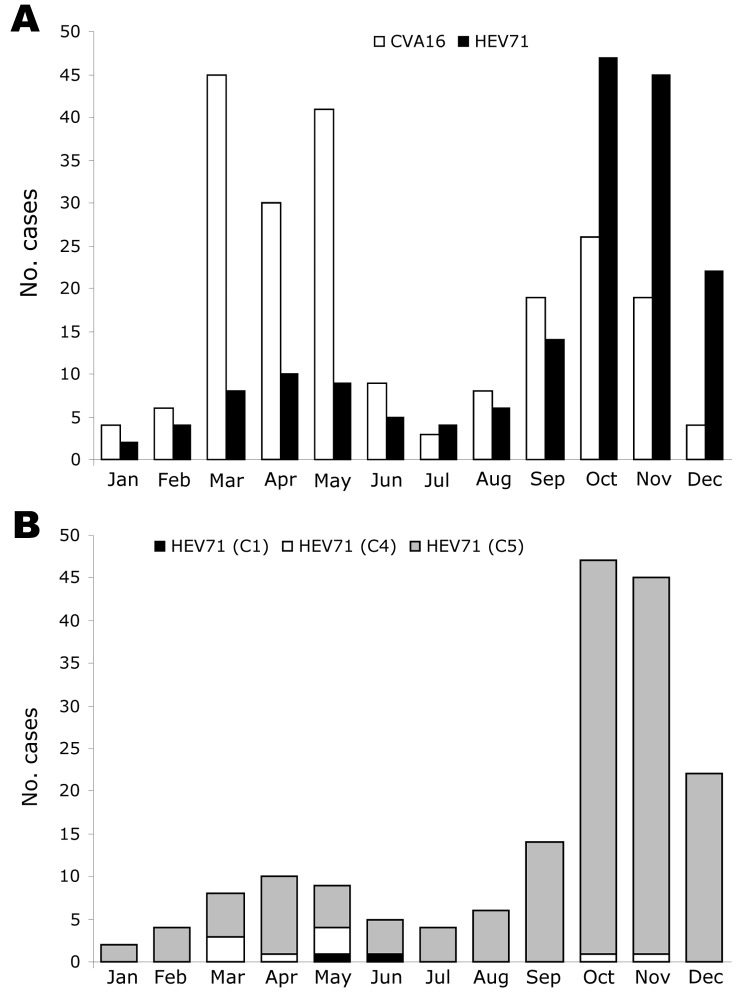
Monthly distribution of 387 cases of hand, foot, and mouth disease (HFMD) associated with isolation of either coxsackievirus A16 (CVA16) (214 cases) or human enterovirus 71 (HEV71) (173 cases), southern Vietnam, 2005. RNA was extracted from cells inoculated with vesicle, throat swab, or stool specimens. Partial VP4 gene sequences were amplified by reverse transcription–PCR (RT-PCR) by using specific primers (*22*), the amplified cDNA sequenced, and the serotype and/or genogroup specificity determined by BLAST analysis. A) Monthly distribution of CVA16 and HEV71-associated HFMD cases. B) Monthly distribution of 173 HFMD cases associated with HEV71 infection with strains belonging to subgenogroups C1, C4, or C5.

The distribution of CVA16- and HEV71-associated HFMD cases by month during 2005 is presented in Figure 3, panel A. HFMD was identified in southern Vietnam throughout the year; HEV71 and CVA16 were also isolated throughout the year. Two peaks of HFMD activity were observed during 2005. The first peak occurred from March through May. CVA16 was the predominant virus during this time, accounting for 81.1% (116 cases) of HFMD compared to 18.9% (27 cases) for HEV71 (Figure 2, panel A). The second peak of HFMD activity occurred from September through December. HEV71 was the predominant virus during this time, accounting for 65.3% (128 cases) of HFMD compared to 34.7% (68 cases) for CVA16 (Figure 3, panel A).

**Figure 4:**
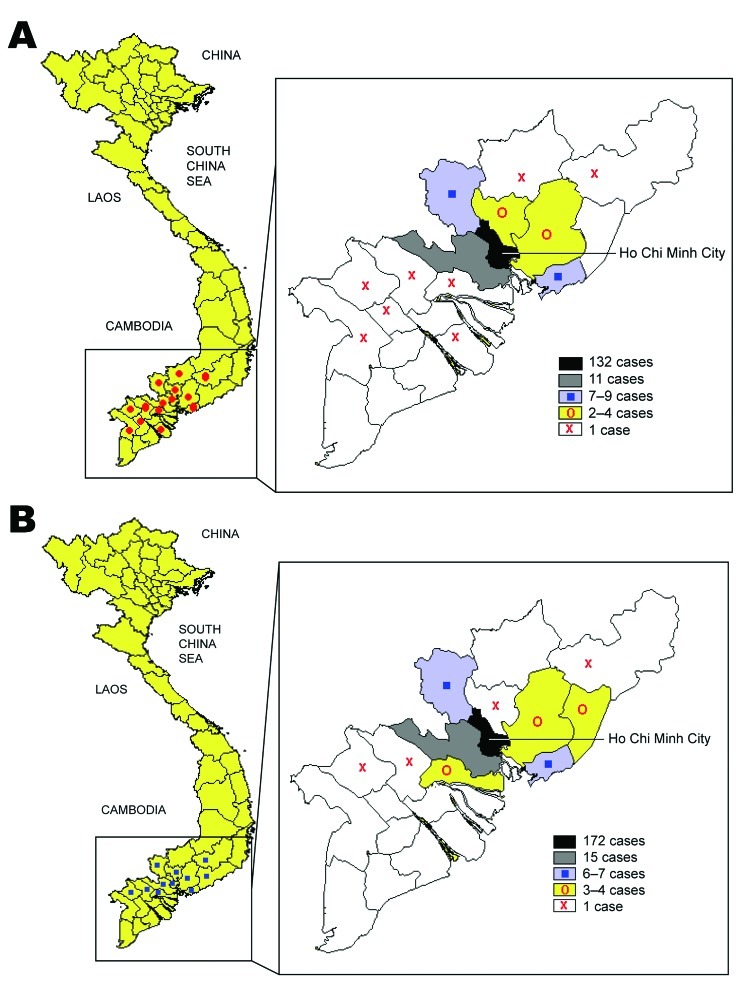
Geographic distribution of hand, foot, and mouth disease cases associated with human enterovirus 71 (A) or coxsackievirus A16 (B) infection, southern Vietnam, 2005.

Figure 4 depicts the geographic distribution of HFMD cases due to HEV71 (Figure 4, panel A) and CVA16 (Figure 4, panel B) who were brought for treatment to a major children’s hospital in Ho Chi Minh City. Children admitted to this hospital are predominantly drawn from the urban area but were also referred from provinces surrounding Ho Chi Minh City.

### Molecular Epidemiology of HEV71

The HEV71 isolates were further analyzed to determine the monthly distribution of viral subgenogroups in southern Vietnam during 2005 (Figure 3, panel B). This analysis was achieved by RT-PCR amplification of complete VP4 and partial VP1 gene sequences, nucleotide sequencing, and BLAST analysis (*20*). Using these methods, we identified 3 HEV71 subgenogroups, C1, C4, and a previously undescribed subgenogroup, C5. Two virus isolates (1.2%) belonging to subgenogroup C1 were identified, 1 each in May and June. A total of 9 (5.2%) subgenogroup C4 strains were identified; 7 were isolated from March through May and 1 each in October and November. Strains belonging to the new subgenogroup C5 (162 [93.6%]/173) were the predominant genetic lineage identified in southern Vietnam during 2005. Subgenogroup C5 viruses were identified in each month and were the primary cause of the large increase in HFMD from September through December.

**Figure 5:**
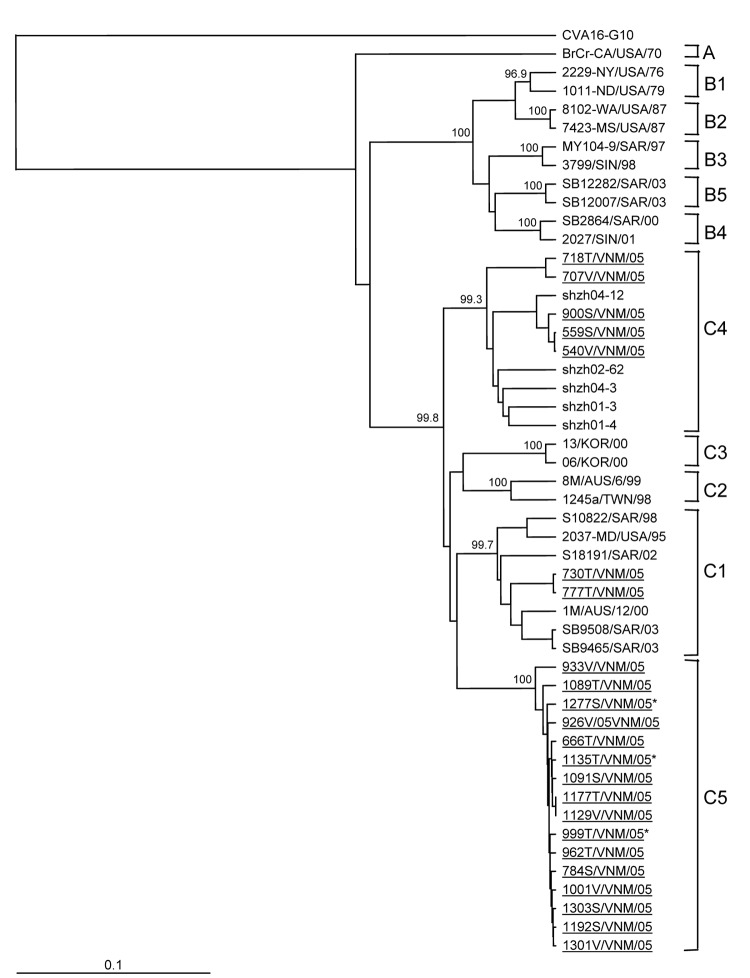
Dendrogram constructed by using the neighbor-joining method (*25*) showing the genetic relationships between 23 human enterovirus 71 (HEV71) strains isolated in southern Vietnam during 2005 (underlined), based on the alignment of complete VP1 gene sequences. Branch lengths are proportional to the number of nucleotide differences. The bootstrap values in 1,000 pseudoreplicates for major lineages within the tree are shown as percentages. The marker denotes a measurement of relative phylogenetic distance. Strain names indicate a unique numerical abbreviation of country and year of isolation. Asterisks (*) denote HEV71 isolates obtained from fatal cases. The prototype coxsackievirus 16 (CVA16)–G10 strain (*28*) was used as an outgroup. The dendrogram shows genogroups A, B, and C as identified by Brown et al. (*24*). Details of the strains used to prepare the dendrogram are shown in Table 1.

Because we had identified a putative new subgenogroup of HEV71 (C5) by analysis of complete VP4 and partial VP1 gene sequences (Figure 3, panel B), we conducted further nucleotide sequence analysis of the complete VP1 gene of 23 HEV71 isolates whose VP4 sequences were representative of all clusters observed in dendrograms generated from the screening data (*9*,*24*). Complete VP1 gene sequence analysis is considered the most rigorous method for determining the molecular phylogeny of HEV71 strains (*6*,*24*), and our analysis needed to be confirmed with a subset of all the isolates (Figure 5). We used previously published VP1 gene cDNA sequences to reconstruct the subgenogroup lineage structure of HEV71, first identified by Brown et al. (*24*) (Table 2).

Two of the Vietnamese HEV71 isolates clustered within subgenogroup C1; 5, within subgenogroup C4; and 16, within the new subgenogroup C5 (Figure 5). The subgenogroup clustering of the HEV71 Vietnamese isolates is strongly supported by bootstrap analysis, which indicates that 3 independent genetic HEV71 lineages (C1, C4, and C5) circulated in southern Vietnam during 2005. This, together with the year-round isolation of CVA16 and HEV71 from HFMD patients (Figure 3, panels A, B), suggests that both viruses circulate endemically in southern Vietnam.

A comparison of the percentage identity of the complete VP1 gene nucleotide sequences of HEV71 subgenogroup C1–4 viruses with that of 16 Vietnamese subgenogroup C5 strains is presented in Table 3. Viruses belonging to subgenogroup C5 shared 89.1%–91.0%, 88.8%–90.1%, 88.8%–89.8%, and 87.7%–90.2% similarity to viruses belonging to subgenogroups C1, C2, C3, and C4, respectively. The consistent 9%–12.3% difference in nucleotide sequence identity between putative subgenogroup C5 strains and those belonging to subgenogroups C1–C4 provides strong evidence for the classification C5 as a new and separate subgenogroup of HEV71.

**Table 3:** Percentage identity of complete VP1 gene nucleotide sequences of HEV71 genogroup C viruses*

Subgenogroup	% Nucleotide identity				
	C1	C2	C3	C4	C5
C1	–	88.4–90.9	89.7–91.2	87.3–91.9	89.1–91.0
C2		–	90.2–91.4	88.7–91.0	88.8–90.1
C3			–	89.3–90.3	88.8–89.8
C4				–	87.7–90.2
C5					–

*HEV71, human enterovirus 71.

## Discussion

To our knowledge, this study provides the first comprehensive epidemiologic and virologic survey of HFMD, CVA16, and HEV71 infection in Vietnam. Similar to the situation in other countries, HEV71 infection was associated with a subset of HFMD cases in which acute neurologic disease developed. Our epidemiologic and phylogenetic data suggest that both CVA16 and HEV71 circulate endemically in southern Vietnam.

Nearly one third of the HEV71-associated HFMD cases identified in our study were complicated by acute neurologic disease. The case-fatality rates of 1.7% in all identified HEV71 infections and 5.9% in HEV71 acute neurologic disease cases are higher than those observed in other studies (*7*,*30*,*31*). However, the case-fatality rates calculated in our study may overestimate the true values because only HFMD patients who were brought for treatment at a major children’s hospital were included in the study. The best estimates of case-fatality rates for HEV71 infection have come from a large seroepidemiologic study of the 1998 HFMD epidemic in Taiwan (*32*); the authors estimated a case-fatality rate of 96.96 per 100,000 population in infants <1 year of age, declining to 6.64 per 100,000 population in children >5 years of age. To rigorously determine the incidence and case-fatality rate of HEV71 infection in southern Vietnam, a similar population-based seroepidemiologic study should be undertaken.

Although cases of HFMD were identified throughout the year, 2 periods of increased prevalence were identified—from March through May and from September through December. In southern Vietnam, these months are interim periods between the dry and wet seasons. CVA16 was the predominant virus isolated in the first period, and HEV71 infection was the predominant virus isolated in the second period. Ongoing epidemiologic surveillance will be necessary to determine whether this pattern of HFMD and enterovirus activity recurs in a regular annual cycle.

Phylogenetic analysis based on nucleotide sequence alignment of the complete VP1 gene of 23 representative strains of HEV71 from southern Vietnam showed that they belonged to 3 subgenogroups, C1, C4, and to the previously undescribed subgenogroup C5. Since 1997, 2 genetically distinct major lineages (B, C) of HEV71 have circulated in different parts of the Asia-Pacific region (*6*,*9*). Viruses belonging to genogroup B have predominated in Southeast Asia, whereas viruses belonging to genogroup C have predominated in northern Asia (*6*,*9*,*11*,*33*). Before 1997, HEV71 strains belonging to subgenogroup C1 were identified in several small outbreaks around the world (*15*,*24*). Since 1997, subgenogroup C1 viruses have circulated endemically in the Asia-Pacific region and have been found to cocirculate as a minor subgenogroup together with a predominant HEV71 subgenogroup during several outbreaks (*6*,*11*,*34*). In this study, subgenogroup C1 viruses comprised only 1.1% of HEV71 strains isolated, indicating low-level circulation. Viruses belonging to subgenogroup C2 have circulated widely in the Asia-Pacific region between 1998 and 2000 (*9*,*11*,*16*) and were responsible for the large outbreak in Taiwan in 1998 (*6*,*8*,*9*,*33*). Two new genetic lineages of genogroup C, subgenogroups C3 and C4, have emerged recently in northern Asia. Viruses belonging to subgenogroup C3 first appeared in the People’s Republic of China in 1998 (*6*) and reemerged in South Korea in 2000 (*6*,*9*). Viruses belonging to subgenogroup C4 were first identified in the People’s Republic of China in 1998 and again in 2000 (*35*) before their identification in southern Vietnam during 2005. Furthermore, a new subgenogroup, C5, circulated widely in southern Vietnam throughout 2005 and became the predominant virus strain identified during the second half of the year.

Our data indicate that the molecular epidemiology of HEV71 in southern Vietnam conforms to the northern Asian epidemiologic pattern of endemic circulation of genogroup C virus strains, with evidence of the ongoing evolution of new subgenogroups, similar to that observed for genogroup B HEV71 strains in Southeast Asia (*6*,*9*,*33*). Furthermore, the year-round isolation and circulation of multiple independent genetic lineages of HEV71 (*36*) suggest that this virus circulates endemically within the human population of southern Vietnam.

In conclusion, this study has established that HEV71 circulates endemically in southern Vietnam and thus represents a substantial threat to the health of children in this region. Improvements in public sanitation and personal hygiene alone are unlikely to prevent HEV71 transmission within the community. A vaccine is necessary to prevent HEV71-induced neurologic disease in susceptible children. However, until such a vaccine is available, virus activity in the community must be monitored through the establishment and maintenance of sentinel surveillance.
